# The diagnostics and laser infrastructure at the Soft X-ray Port of the European XFEL

**DOI:** 10.1107/S1600577526005886

**Published:** 2026-07-17

**Authors:** Patrik Grychtol, Vahagn Vardanyan, Pranav Bhardwaj, Michael Heber, David Doblas-Jimenez, Ekaterina Tikhodeeva, Serguei Molodtsov, Manuel Izquierdo

**Affiliations:** a European XFEL Holzkoppel 4 22869Schenefeld Germany; b Deutsches Elektronen-Synchrotron DESY Notkestraße 85 22607Hamburg Germany; c Institute of Experimental Physics TU Bergakademie Freiberg Leipziger Strasse 23 09599Freiberg Germany; d Center for Efficient High Temperature Processes and Materials Conversion (ZeHS) TU Bergakademie Freiberg Winklerstrasse 5 09599Freiberg Germany; European XFEL, Germany

**Keywords:** Soft X-ray Port (SXP), European XFEL, time-resolved X-ray photoelectron spectroscopy (tr-XPES), pump–probe spectroscopy, instrument response function (IRF)

## Abstract

The diagnostics and laser infrastructure of the Soft X-ray Port (SXP) at the European X-ray Free Electron Laser are presented. Designed as a supplementary open port, SXP aims to enhance the research capabilities alongside the existing baseline instruments at the SASE3 soft X-ray undulator exploiting the high repetition rate operation enabled by superconducting acceleration cavities. These instruments already support studies in atomic, molecular and non-linear optics (SQS), as well as condensed matter physics (SCS). The inception of SXP is largely motivated by the community involved in time-resolved X-ray photoelectron spectroscopy (tr-XPES), focusing on material science dynamics at surfaces and interfaces. The dedicated endstation equipped with a time-of-flight momentum microscope allows for simultaneous recording of energy- and angle-resolved photoelectron distributions in a pump–probe setting, employing two synchronized femtosecond laser systems meant to cover a broad spectrum from the infrared to the extreme ultraviolet region. The alignment laser system, pump–probe laser transport, including the in-coupling optics, and the installed photon arrival-time monitor are described. Under the present operating conditions, however, the photon arrival-time monitor does not yet provide routine single-shot timing corrections, so the currently demonstrated temporal benchmark is obtained *in situ* at the tr-XPES endstation. Measurements on a patterned Au reference sample show that the laser spot sizes can be characterized directly and that sidebands yield an X-ray/optical cross-correlation of about 58 fs (FWHM). These measurements define the spatial and temporal instrument response currently achieved for pump–probe experiments at SXP.

## Abstract 2

The diagnostics and laser infrastructure of the Soft X-ray Port (SXP) at the European X-ray Free Electron Laser are presented.

## 1. Introduction

The development of X-ray free-electron laser (XFEL) facilities, capable of generating extremely brilliant photons with energies in the kilo-electronvolt (keV) range, have revolutionized the ability to probe the nanocosm of atoms, molecules and condensed matter (McNeil & Thompson, 2010 ▸). Their energy-tunable, ultrafast and highly coherent photon pulses enable detailed, site-specific exploration of light–matter interactions on fundamental timescales encompassing nuclear and electronic motions (Feldhaus *et al.*, 2013 ▸; Bostedt *et al.*, 2016 ▸). A second revolution in the field of XFELs has been the increase in repetition rate enabled by superconducting accelerating radiofrequency (RF) cavities. First demonstrated at FLASH in the soft X-ray regime, the first hard X-ray implementation was realized at the European XFEL (Decking *et al.*, 2020 ▸). This new capability has widely enlarged scientific possibilities, enabling unprecedented experiments from the soft to the hard X-ray range (Wiedorn *et al.*, 2018 ▸; Büttner *et al.*, 2021 ▸; Shvyd’ko *et al.*, 2023 ▸; Nowakowski *et al.*, 2024 ▸; Richard *et al.*, 2025 ▸; Kraus *et al.*, 2025 ▸; Su *et al.*, 2026 ▸). It also allows for the extension of tr-XPES experiments, previously pioneered at FLASH, into the soft X-ray range available at the SASE3 undulator of the European XFEL.

In the realm of time-resolved experiments, the widely adopted pump–probe technique involves exciting the target system with a *pump* pulse, followed by probing its subsequent response with a temporally displaced *probe* pulse. When these pulses originate from disparate sources, such as an accelerator-based XFEL and an optical laser (OL), the temporal resolution is constrained by pulse duration and timing jitter. Hence, achieving the utmost time resolution in pump–probe experiments necessitates both the generation of ultrashort laser pulses and the meticulous synchronization thereof. Furthermore, to exploit site-specificity and comprehensively address electronic, magnetic and phononic degrees of freedom in time-resolved experiments, it becomes imperative to dynamically tune the investigative photon sources across a wide wavelength range. This should encompass full control over the polarization and ideally extend to the entire electromagnetic field of the ultrafast photon pulses.

At the Soft X-ray Port (SXP) of the European XFEL, these general requirements are realized by an integrated laser infrastructure designed for user operation. A central pump–probe laser provides two synchronized outputs (centered around 800 nm in the femtosecond regime and 1030 nm in the picosecond regime) with operating points matched to the facility’s burst-mode timing structure. The 1030 nm branch can even be compressed to sub-50 fs durations, and an additional high-repetition-rate Yb-fiber laser amplifier is available, both routing toward the planned wavelength extension via the generation of higher harmonics and optical parametric amplification schemes. Single-shot timing between OL and XFEL pulses is envisioned to be characterized and correctable via a photon arrival-time monitor, enabling post facto jitter mitigation. OL beam transport to the endstation is implemented through a dedicated in-coupling and delivery system that provides a collinear geometry with the XFEL, adjustable delays, fluence control and flexible focusing onto the sample under investigation. Together, these elements furnish the tunability (from IR into the UV/XUV via frequency conversion and high harmonic generation), intensity control, and synchronization needed for ultrafast pump–probe studies at SXP under realistic user conditions.

In the following, this contribution presents the conceptual framework and first results for the diagnostics and laser infrastructure of SXP at the European XFEL (Tschentscher *et al.*, 2017 ▸). Designed as a supplementary open port, SXP aims to enhance the research capabilities alongside the existing baseline instruments at the SASE3 soft X-ray undulator (Izquierdo, 2022 ▸). These instruments already support studies in atomic, molecular, and non-linear optics (SQS – Small Quantum Systems) (Meyer, 2022 ▸), as well as condensed matter physics (SCS – Spectroscopy and Coherent Scattering) (Carley *et al.*, 2022 ▸). The inception of SXP is largely motivated by the community involved in tr-XPES, focusing on material-science dynamics at surfaces and interfaces employing a time-of-flight (ToF) momentum microscope (MM) (Aeschlimann *et al.*, 2025 ▸; Kutnyakhov *et al.*, 2020 ▸). Furthermore, SXP opens avenues for exploring high-valent metal intermediates in biological and inorganic catalysts through fluorescence spectroscopy and delves into the study of highly charged ions with regard to astrophysical phenomena. The European XFEL’s exceptional features enable SXP to conduct unique ultrafast pump–probe experiments, leveraging powerful and adjustable soft X-rays in conjunction with synchronized femtosecond laser systems meant to cover a broad spectrum from the infrared (IR) to the extreme ultraviolet (XUV) region (Grychtol *et al.*, 2022*a* ▸; Grychtol *et al.*, 2022*b* ▸).

## 2. Overview of SXP

SXP is positioned after the SASE3 soft X-ray undulator offering photons with adjustable polarization across an energy spectrum from about 260 eV to 3000 eV, with intensities surpassing 10^12^ photons per pulse with up to 27000 pulses per second. Utilizing two bendable, high-precision elliptical mirrors in a Kirkpatrick–Baez (KB) configuration, it can focus femtosecond XFEL pulses to a spot between around 2 µm and 5 µm in diameter, depending on the position of the experimental station at the end of the SXP beamline, thus achieving intensities up to 10^18^ W cm^−2^ in the target area. This infrastructure is complemented by several synchronized femtosecond laser systems, capable of extending their wavelength coverage into the IR and XUV regions, thereby enabling ultrafast pump–probe experiments that merge the power of intense, tunable soft X-rays with the flexibility of optical lasers.

**Figure 1:**
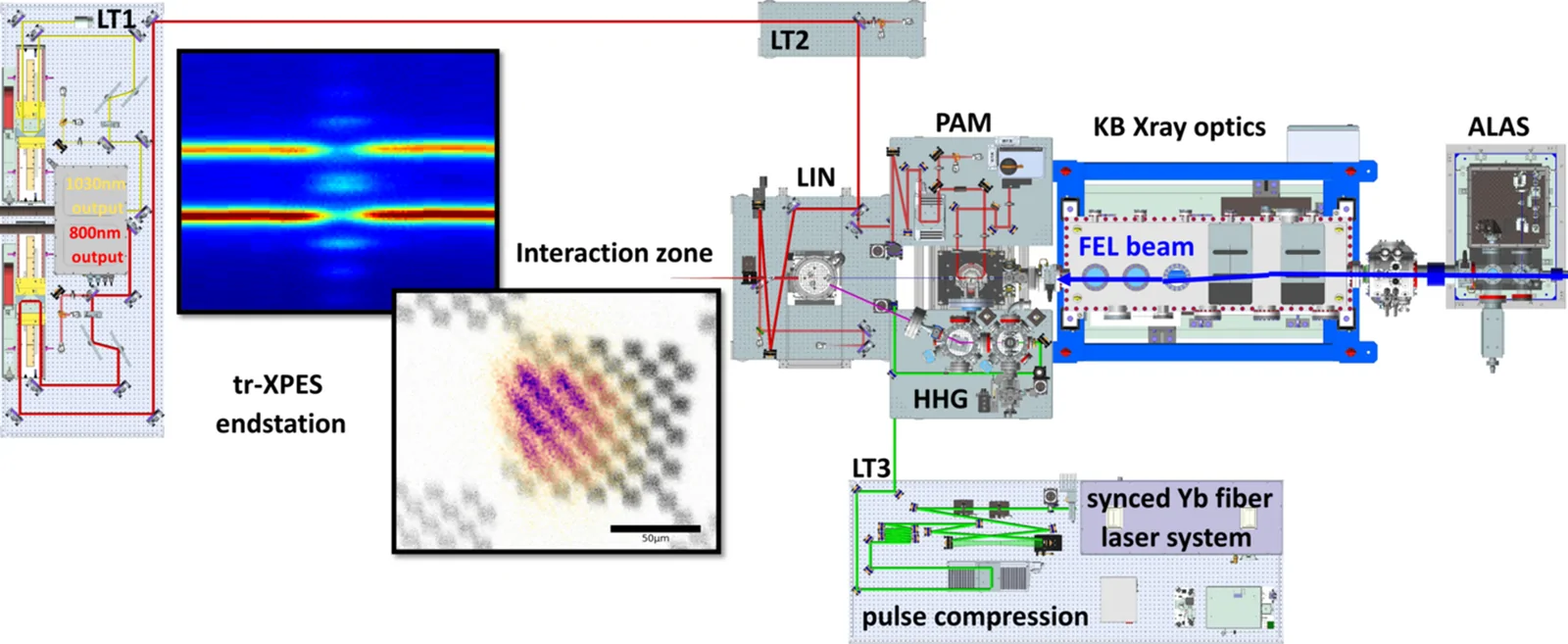
Top-view schematic of the SXP mechanical model showing the major permanent beamline components (experimental endstation not included). Exemplary 800 nm and 1030 nm optical-laser beam paths are indicated for the central pump–probe laser (not shown; entering from the left on laser table LT1) and the stand-alone Yb-fiber amplifier system on laser table LT3 (bottom). The XFEL beam enters from the right, traverses the alignment laser system (ALAS) and the X-ray optics chamber, and is subsequently routed through the photon arrival time monitor (PAM) and the laser in-coupling unit (LIN) before being focused into the interaction zone.

Fig. 1 ▸ illustrates a top view of the SXP mechanical model, excluding an experimental endstation, but comprising the major permanent beamline components. The diagram provides an exemplified representation of the 800 nm and 1030 nm laser beam paths originating from the central pump–probe laser (not shown here – entering the figure on the left-hand side on laser table LT1) and the stand-alone Yb-fiber laser amplifier system placed on laser table LT3 (bottom of the figure). The XFEL beam enters SXP from the right-hand side of the figure, traversing the alignment laser system (ALAS) before entering the KB optics chamber. Thus, the soft X-ray beam is routed through the photon arrival time monitor (PAM) and the laser in-coupling unit (LIN) before it is focused into the interaction zone. In this sequence, the PAM serves the purpose of measuring the relative arrival times between XFEL and OL pulses, while the LIN facilitates the co-linear coupling of the OL beam into the XFEL path. This alignment ensures that both beams can be accurately focused onto the sample, also minimizing the geometric timing jitter.

## 3. ALAS – the alignment laser system

**Figure 2:**
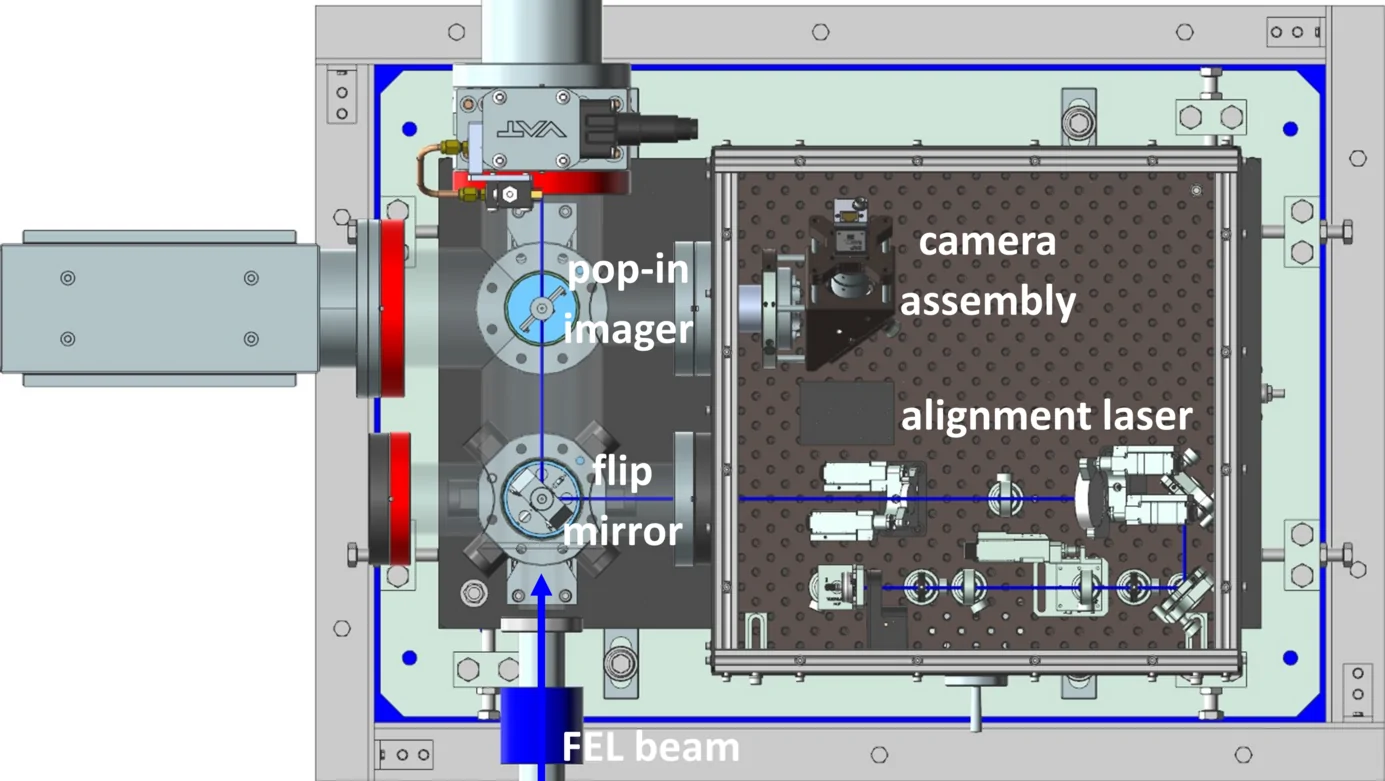
Top view of the mechanical layout of the alignment laser system (ALAS) positioned upstream of the KB X-ray optics chamber. The XFEL beam enters from below (CF 40 flange) and traverses two four-way-cross sections with motorized manipulators: A flip mirror injects an optical alignment laser parallel to the XFEL path, while pop-in fluorescent screens can be inserted to monitor both beams.

The ALAS is an advanced optical laser and imaging setup designed to streamline and enhance the alignment of downstream beamline components. Its main function is to facilitate the precise positioning of the SXP components, ultimately conserving crucial beam time, minimizing the risk of beam damage, and reducing wear on components susceptible to intense XFEL radiation. A mechanical representation of the ALAS, positioned directly before the KB X-ray optics chamber, is illustrated in Fig. 2 ▸. In this depiction, the XFEL beam is introduced into the ALAS chamber from below via a CF 40 flange, traversing through two four-way-cross structures, each of which accommodates a manipulator. The initial manipulator incorporates a drop-in mirror to direct an optical laser beam parallel to the XFEL beam path, whereas the subsequent manipulator contains a set of fluorescent screens for tracking the alignment of both laser beams. This system, in conjunction with extra pop-in screens situated further along in the PAM or LIN sections, enables precise alignment of SXP without the need for active XFEL operation.

Within the black box, positioned at a right angle to both manipulators, lies the laser diode module (LASOS – BLK 7310 TS) equipped with alignment optics and a CCD camera (Basler acA2040-35gm) assembly. For the ALAS to be most effective, it is essential that it not only travels in alignment with the XFEL beam but also replicates its profile and divergence. This capability enables the fine-tuning of the KB mirrors to focus on the sample using solely an optical beam. For this purpose, a telescope can be positioned after the laser diode to expand the optical beam to match the dimensions and divergence of the X-ray beam. All components are motorized and can be remotely operated in order to efficiently align the beamline. A more detailed description and characterization of its performance will be described elsewhere.

## 4. LAS – the pump–probe laser system

### 4.1. The pump–probe laser specifications

**Figure 3:**
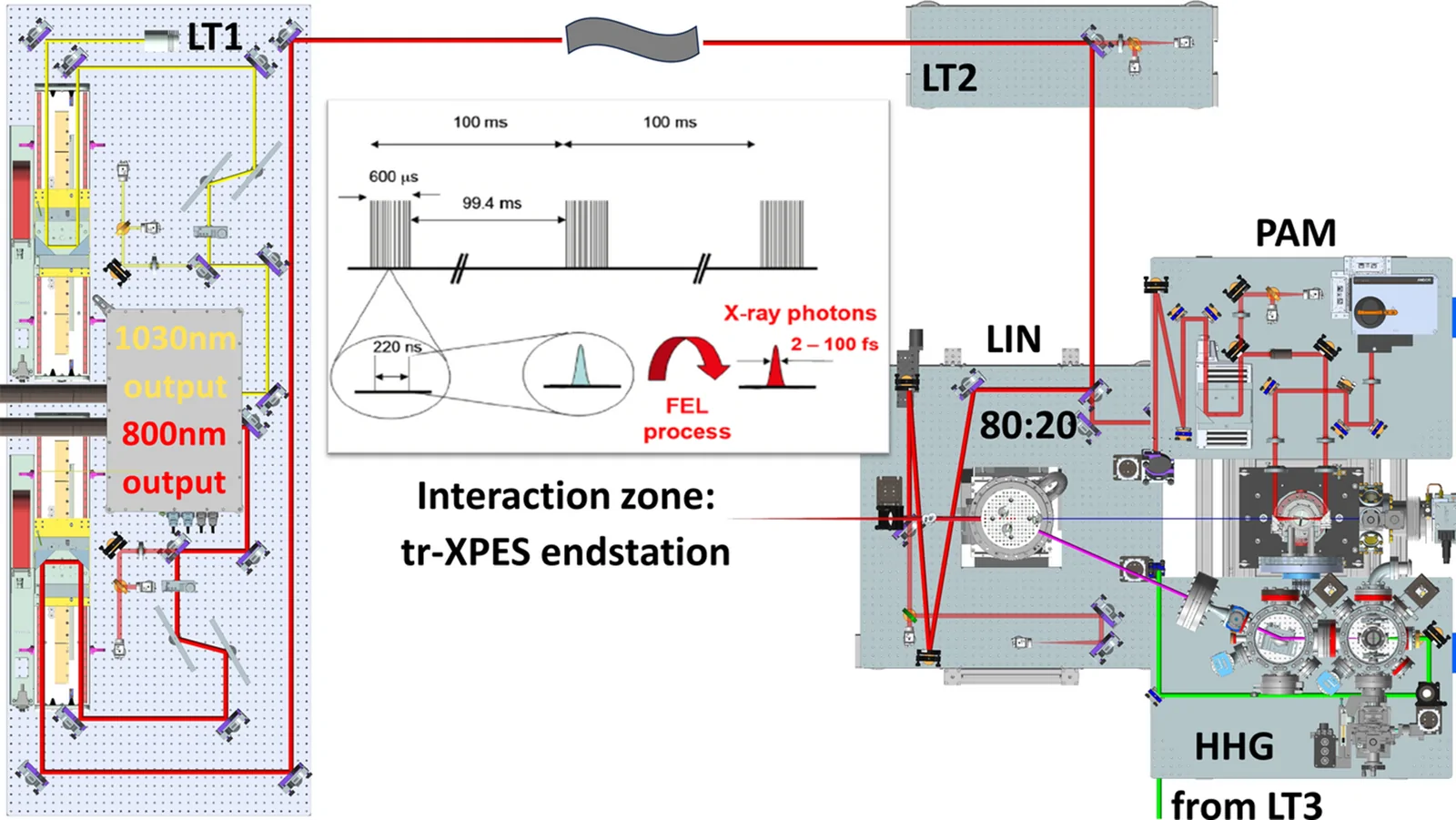
Overview of the 800 nm and 1030 nm beam paths from the central pump–probe laser entering the SXP hutch on the laser table LT1, where both beams can be attenuated and delayed in time with respect to the FEL beam. They are then routed via LT2 to the laser in-coupling system (LIN) and photon arrival time monitor (PAM) with a splitting ratio of 80:20.

Ultrafast optical pulses at SXP are primarily supplied by the central pump–probe laser developed by the European XFEL Laser (LAS) group (Pergament *et al.*, 2016 ▸). This laser operates in a 20 Hz burst mode with an intra-burst pulse structure that mirrors that of the European XFEL, as shown in the inset of Fig. 3 ▸. The 20 Hz operation enables the beam to be sent simultaneously to the two baseline experiments at SASE3. Consequently, both instruments (SQS and SCS) can function at 10 Hz with the same pattern as the X-ray pulses. Although it is planned to offer laser pulses across a wide range of wavelengths around the visible spectrum through various frequency conversion schemes, in the current operational configuration relevant for SXP user experiments, primarily two beams can be delivered to the SXP experiment hutch: one centered at 800 nm with pulse durations in the femtosecond range (nearly bandwidth limited from about 15 fs to 300 fs), and the other centered at 1030 nm with pulse durations in the picosecond range (mainly 0.9 ps and 400 ps), which can be compressed to around 40 fs via a Herriott-type multi-pass cell (HMPC). Both beams are routed through the dedicated instrument laser hutch (ILH) and enter the SXP experiment hutch via an elaborate delivery system starting on laser table LT1, as depicted on the left side of Fig. 1 ▸. The SASE3 central pump–probe laser has several primary set points with an intra-burst pulse pattern matching that of the XFEL, which are summarized in Table 1 ▸.

Both laser beams allow for optical excitations at discrete wavelengths in the UV region by frequency up-conversion through second-, third- and fourth-harmonic generation (SHG/THG/FHG) reaching as low as 257 nm. In the case of the 800 nm beam, however, fourth-harmonic generation is not feasible at these high pulse energies, because absorption of the generated light by the conversion crystals leads to its rapid degradation. To fill the spectral gaps until about 250 nm, an optical parametric amplifier (OPA) system has been successfully commissioned by the laser group, which at the same time allows for tuning the wavelength into the mid-IR region until approximately 15 µm to enable resonant excitations of collective low-energy modes, such as phonons and magnons (Pergament *et al.*, 2016 ▸). However, the high repetition rates required to efficiently perform tr-XPES restrict the use of the OPA system, since the frequency conversion efficiency is inherently low (a few percent) limiting the available amount of pump intensity on target. At the same time, this requirement also reduces the range of usable repetition rates, displayed in Table 1 ▸, to the MHz regime with the current maximum RF window of 600 µs confining the number of pulses to a maximum of about 2700 per 10 Hz bunch train. This results in a maximum effective repetition rate of 27 kHz for any pump–probe experiment at European XFEL. However, since this RF window is shared among three undulators, this reduces to about 8 kHz, when the intra-bunch repetition rate is set to 4.5 MHz.

In addition to the SASE3 pump–probe laser, SXP is equipped with a Yb fiber laser system centered at 1030 nm, delivering 250 fs short and 200 µJ strong laser pulses at a repetition rate of about 334 kHz (Active Fiber Systems, Jena). An internal acoustic-optic modulator (AOM) allows for mimicking the characteristic 10 Hz bunch pattern, and its pulse train can be fully synchronized to the European XFEL (Schulz *et al.*, 2015 ▸). A recent development effort has allowed for compressing the pulse duration to below 40 fs using a Herriott-type multi-pass cell (Viotti *et al.*, 2022 ▸; Grychtol *et al.*, 2023 ▸). Using this laser that has been placed on laser table LT3 and allocated as back-up for the central pump–probe laser, it is also planned to extend the wavelength range into the XUV region employing high harmonic generation (HHG) techniques (Grychtol *et al.*, 2025 ▸). Thus, it will be possible to operate SXP more efficiently by allowing for additional beam time preparation and research opportunities.

### 4.2. The pump–probe laser beam paths

Fig. 3 ▸ sketches more closely the delivery of the 800 nm and 1030 nm outputs entering from the ILH into the SXP experimental hutch, which are eventually split into a sample-pump branch and a timing-diagnostics branch on the LIN laser table. Both wavelengths follow equivalent beam conditioning and diagnostics steps, with minor wavelength-specific optical components, such as the dedicated low dispersive, broadband, high-reflectivity, dielectric mirrors (LAYERTEC) used throughout the respective beam paths.

After admission of the laser beams through the interlocked Amphos laser safety shutter, each wavelength is routed through the first laser table (LT1). Here, attenuation is set using a polarization-based attenuator, an achromatic half-wave plate (B. Halle) combined with two broadband thin-film polarizers (TFPs) to achieve the desired on-sample fluence without altering upstream source settings. The pump–probe delay is introduced on this table by a motorized linear stage (Steinmeyer PLT165-DLM) carrying two mirrors aligned in a retro-reflector geometry (double pass). The stage has 500 mm travel with micrometre-level positioning accuracy and an encoder resolution in the nanometre range. The corresponding optical delay is approximately 6.67 ps mm^−1^, yielding a total scan range of about 3.3 ns. In practice, the minimum granularity is set by overall timing stability rather than the mechanical resolution, to be discussed further below.

At selected steering mirrors positions, about 0.1% of the main beam transmitted from the high reflectivity surface is imaged onto a pair of ‘leakage cameras’ (Basler acA2040-35gm). These near-field views provide continuous monitoring of beam shape and pointing for both wavelengths. Calibration uses the 3.5 µm pixel pitch and a reference target, enabling quantitative pointing analysis and drift correction using sets of upstream motorized mirror mounts that can be remote controlled.

From the first table, the beams are transferred through enclosed beam tubes to the small laser table (LT2), which serves as a relay station and provides space for optional additional conditioning—such as the rotation of polarization, frequency conversion (*e.g.* SHG/THG/FHG) or further attenuation/beam-sizing optics—without altering the overall delivery geometry. They are then passed onto the LIN laser table, where the pump/diagnostics division and the sample focusing occur, while the beam height is adjusted to the XFEL level by means of polarization-preserving periscopes. A beam-splitting mirror routes 80% of the power to the sample-pump branch and 20% to the timing-diagnostics branch that feeds the photon arrival-time monitor.

## 5. PAM – the photon arrival time monitor

**Figure 4:**
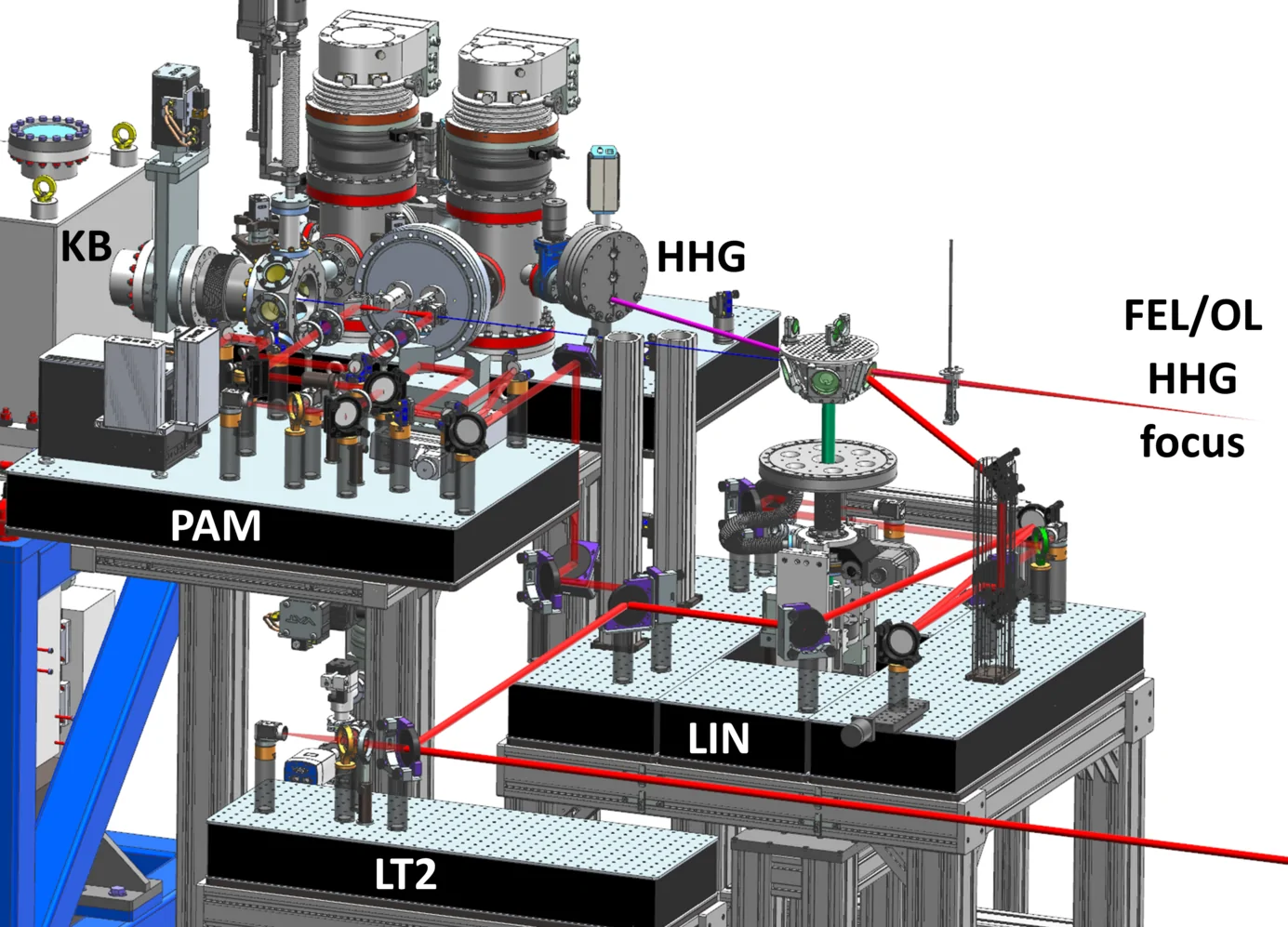
Close-up view of the PAM and LIN showing the optical laser beam paths. Some mechanical components in this figure have been omitted for the sake of illustration.

In order to achieve the highest possible time resolution in pump–probe experiments that combine an OL with an XFEL, it is essential not only to generate ultrafast laser pulses but also to synchronize these light sources properly and compensate for any residual jitter between them. By using a dedicated balanced optical cross-correlation technique based on the actively stabilized distribution of an optical reference signal, a relative in-loop timing jitter at the sub-10 fs level has been achieved at the European XFEL (Schulz *et al.*, 2015 ▸). A practical method to mitigate any residual jitter involves measuring the relative arrival time of the pump and probe pulses on a single-shot basis and sorting the data accordingly in the subsequent analysis process, potentially achieving sub-femtosecond accuracy (Harmand *et al.*, 2013 ▸). For this purpose, SXP is equipped with a PAM located downstream of the KB soft X-ray focusing system. As illustrated in Fig. 4 ▸, it receives a weak portion of the OL pulses split off from the main beam used to pump the sample under investigation. After passing through a delay stage (Physik Instrumente HPS-170), these pulses are stretched in time by propagating through a thick piece of glass (Schott SF57) and are then focused onto a thin semi-transparent membrane, such as Si_3_N_4_, which is simultaneously illuminated by the soft X-ray beam. The XFEL pulses alter the refractive index and thus the transmission of the membrane material, whose transient response is measured by the chirped OL pulses, mapping their relative delays to the soft X-ray pulses onto the optical laser spectrum monitored with a high resolution spectrometer (Andor Kymera). This established technique is known as spectral encoding (Bionta *et al.*, 2014 ▸).

The PAM was designed and implemented by the X-ray photon diagnostics group at the European XFEL (Liu *et al.*, 2017 ▸). It has been successfully used to monitor the relative arrival times between XFEL and synchronized OL pulses, measuring a timing jitter of approximately 58 fs (FWHM) at SASE3. While it has been demonstrated that applying these time-of-arrival corrections in pump–probe experiments can significantly enhance their temporal resolution (Grychtol *et al.*, 2021 ▸; Rivas *et al.*, 2022 ▸), the present geometry and intensity conditions at SXP have not yet yielded a detectable XFEL-induced modulation of the PAM membrane. This outcome reflects the combination of (i) the mandatory placement of the PAM between KB and endstation imposed by extremely limited hutch space that results in an FEL beam quite elongated in the horizontal direction (1.4 mm), and (ii) the low-fluence operating regime of the tr-XPES endstation to avoid space-charge and sample-damage effects. The typical, effective FEL spot size at the PAM imager was measured to be around 0.5 mm (FWHM). This results in a maximum peak fluence of 0.3 mJ cm^−2^, well below the 5 mJ cm^−2^ threshold required for efficient operation, assuming a FEL pulse duration of about 30 fs at 1 keV photon energy. Together, these factors limit the soft X-ray intensity available at the transducer at the more or less fixed PAM location.

Accordingly, although the PAM hardware and optical branch are installed at SXP and the spectral-encoding approach is well established at European XFEL, the present implementation does not yet provide a usable XFEL-induced signal under the low-fluence operating conditions typical for current tr-XPES experiments. Routine single-shot arrival-time correction is therefore not presently available for user operation at SXP. At this stage, the experimentally demonstrated temporal benchmark for SXP pump–probe measurements is obtained *in situ* at the endstation via laser-assisted photoelectric (LAPE)-based cross-correlation. Nonetheless, the present results at SXP provide a clear design target for an upgraded timing diagnostic tailored to low soft X-ray fluences. The mechanical, optical and control infrastructure already in place (separate diagnostics branch, independent attenuation and delay, versatile vacuum chamber, proximity to LIN/KB, synchronized readout) facilitates rapid iteration. Commissioning of a revised arrival-time monitor will be reported separately; in the meantime, the guidance presented in this contribution enables timing-aware planning and analysis for tr-XPES and related low-fluence, high repetition rate experiments at SXP.

## 6. LIN – the laser in-coupling

The LIN chamber is the most downstream component of SXP, positioned directly upstream of any experimental endstation. Its function is to couple the OL pump beam collinearly into the XFEL path and to provide the last alignment, diagnostics and focusing interfaces before the sample, which is accomplished by a curved mirror system located outside the chamber. Thus, the geometric timing jitter and chromatic aberrations are minimized also keeping transmissive elements out of the vacuum beam path.

The OL beams arrive at the LIN table from LT2/3 and are eventually brought to the XFEL beam height by a polarization-preserving periscope. Just like on all laser tables upstream, all steering and conditioning optics on the LIN table use low-dispersion, broadband dielectric mirror coatings matched to the working wavelength to minimize additional group delay dispersion. Where UV operation is required after frequency conversion, dedicated coatings are inserted in the same mounts without changing the beam geometry.

Inside the LIN chamber, a two-story rotatable carousel holds multiple 2-inch mirrors at 15° incidence, each with a central aperture of 2.7 mm that allows the XFEL to pass while reflecting the OL. Vacuum-compatible, piezo-motor-driven mirror mounts provide nanometre-precision pointing for reproducible collinear coupling (SmarAct – STT-50.8). Mirror choices (coating, substrate) are selected per experiment to balance reflectivity, bandwidth and damage threshold in order to cover a wide range of science cases without having to break vacuum. The annular aperture imposed by the central hole does not affect the XFEL and has a negligible impact on the OL wavefront at the standard focusing conditions used at SXP.

Downstream of the holey in-coupling mirrors, a variety of scintillators on a manipulator can be moved into the beam path to image the OL and XFEL beams for spatial overlap, facilitating the alignment of the entire beamline when used with the ALAS. Additionally, the manipulator is equipped with an ultrafast photodiode (Thorlabs FDS015) to refine the temporal overlap of both beams into the 100 ps range when coupled with the 12.5 GHz oscilloscope (Tektronix DPO 71254C) of SXP.

Ray-tracing simulations conservatively estimate that using a pair of curved mirrors placed on the optical table below the LIN chamber can achieve a focus with a diameter of less than 100 µm (FWHM) at the sample interaction region. This translates to an intensity exceeding 10^15^ W cm^−2^ mJ^−1^ pulse energy on the target using the 800 nm beam with a pulse duration of 15 fs. For the pump–probe measurements discussed below, the on-sample optical fluence was estimated from pulse-energy measurements performed right after the focusing optics using a calibrated power meter [Ophir 50(150)A-BB-26-V1], corrected for the transmission of the delivery optics between that diagnostic point and the sample. These corrections included the applied attenuation setting and the transmission/reflectivity of the relevant optics in the downstream LIN beam path. The effective illuminated area on the sample was derived from the measured optical focus dimensions and the projection onto the sample surface at the stated incidence geometry. The quoted fluence values should therefore be understood as best estimates of the on-sample fluence rather than direct calorimetric measurements at the sample position. The uncertainty is dominated by the pulse-energy calibration, shot-to-shot fluctuations, transmission estimates and spot-size determination, amounting to approximately 5%.

In order to monitor the size and alignment of the OL beam during a running experiment, two leakage cameras have been installed in a transmission (>1%) geometry of the 45° mirror that is steering the beam into the periscope located downstream of the curved mirror optics. While the first camera is positioned close to the leakage mirror in the near field, the second camera has been mounted on a linear translation stage and thus can be comfortably moved into the focus position.

**Figure 5:**
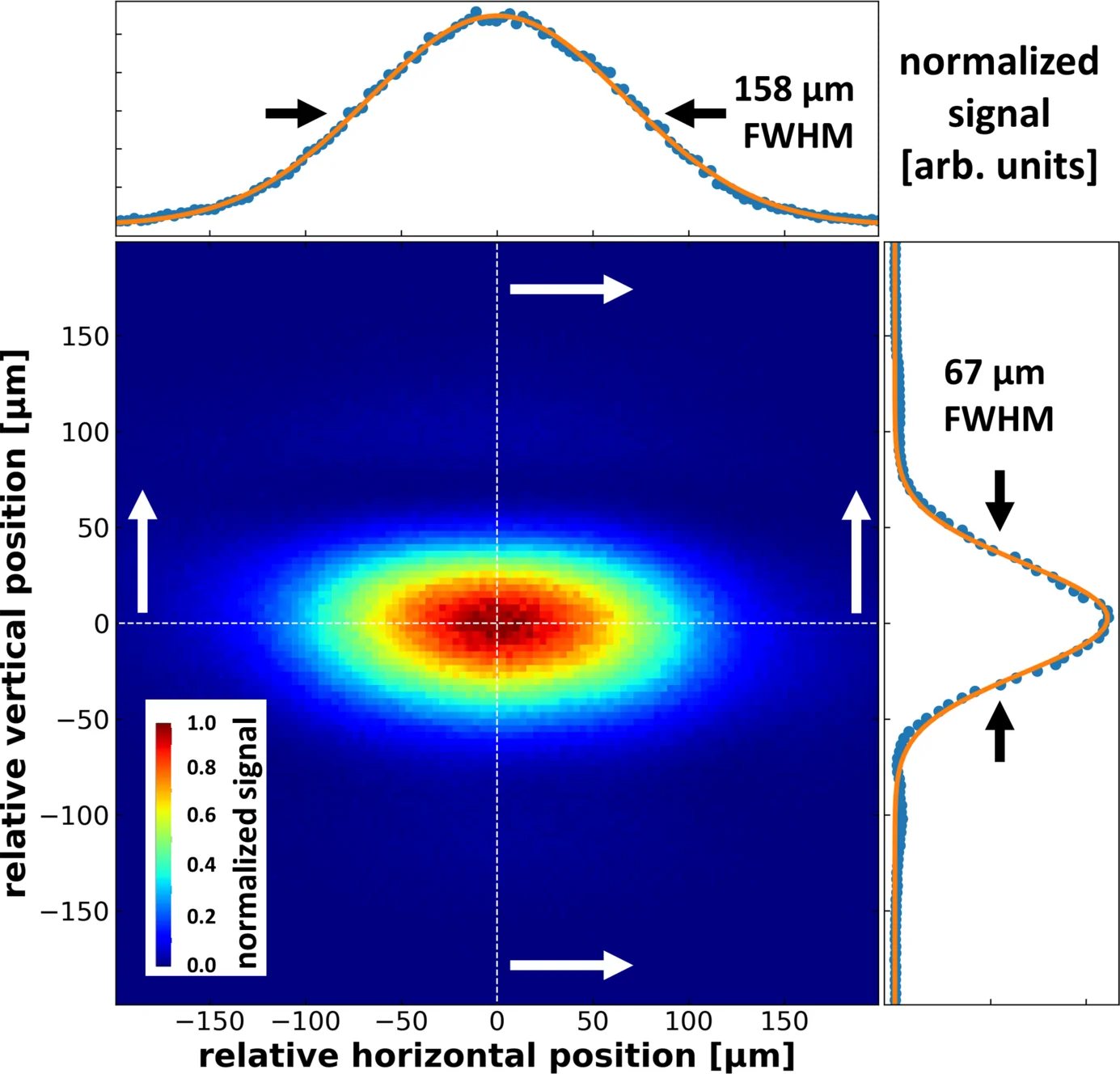
OL focus characterization from the leakage camera after the focusing optics, elongated along the horizontal axis to compensate vertical tilt of the sample.

**Figure 6:**
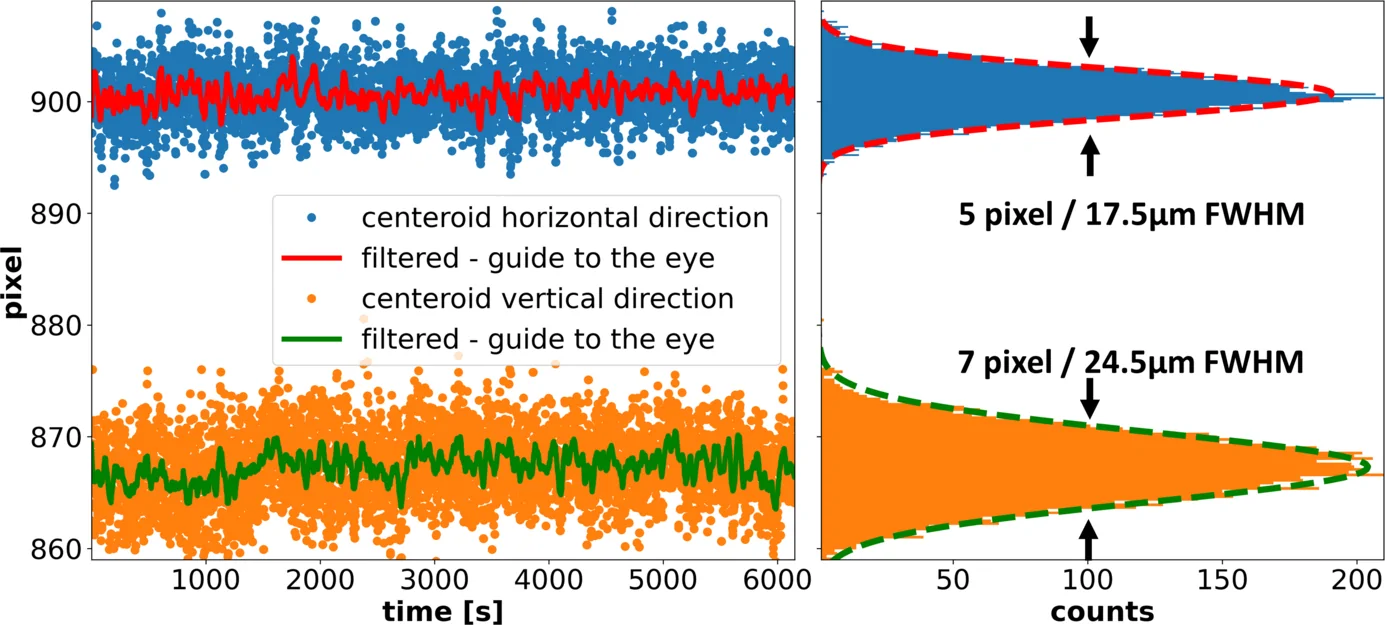
Shift of the OL focus centroid position over the course of an hour.

As can be seen in Fig. 5 ▸, which shows an image from the second leakage camera, an average focus size (FWHM) of approximately 113 µm has been measured, whose center of mass jitters in a period of an hour by about 20 µm (FWHM), as visualized in Fig. 6 ▸. In order to obtain an approximately circular focal spot on the sample, which is tilted by about 68° with respect to the FEL/OL propagation axis, the incidence angles of the spherical focusing mirror assembly was increased such that the optical focus is elongated by roughly a factor of two along the horizontal axis before projection.

## 7. IRF – the instrument response function

The spatial and temporal instrument response function (IRF) at SXP can be characterized at the tr-XPES endstation, a time-of-flight momentum microscope performing photoemission electron microscopy (PEEM) and exploiting the LAPE effect on a well defined metallic reference sample (PLANO – Chessy). It is a polycrystalline gold test structure on a silicon substrate, originally designed for magnification calibration in scanning electron microscopy (SEM). More than 1.6 million gold squares with 1 µm edge length form a fourfold nested chessboard pattern with an overall active area of 5 mm × 5 mm (total chip size 1 cm^2^). The smallest chessboard is 10 µm × 10 µm, which builds up to 100 µm × 100 µm, then 1 mm × 1 mm, and finally fills the 5 mm × 5 mm field, surrounded by a 10 µm wide frame. The dimensional accuracy is better than 200 nm and the orthogonality better than 1 arcsec, making this structure well suited for calibrating magnification, checking image distortions, and estimating spatial resolution at the edges of the gold squares.

For the IRF measurements and the preparation of any pump–probe experiment, a pristine Chessy sample is inserted into the MM and mounted into its custom-designed cryo-cooled hexapod holder that is installed at an incidence of about 68° with respect to the FEL/OL laser beam direction. The MM is equipped with a cathode-lens objective and a long drift tube pointing at normal incidence on the sample, which allows for simultaneous recording of energy- and 2D spatial photoelectron distributions using a delay-line detector (Surface Concept DLD8080). Depending on the setting of the electron optics, the spatial information of the detector can be used to map either the angular distribution (momentum mode) or the real space (PEEM).

**Figure 7:**
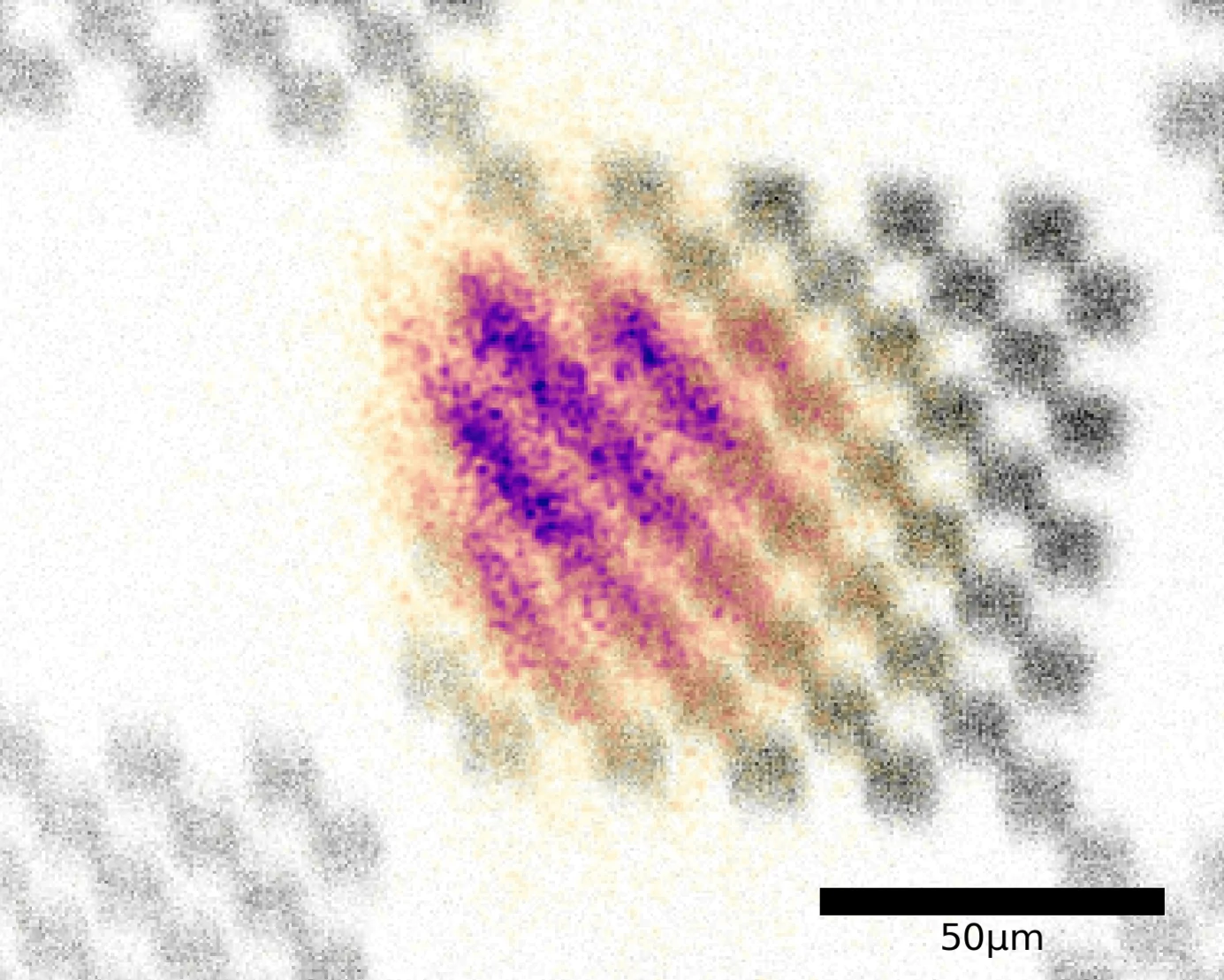
PEEM image of an Au chess patterned reference sample using an Hg-discharge lamp (black/white colors) superimposed with the FEL (purple/orange colors).

In PEEM (imaging) mode, this microscope can be used to map the spatial intensity profile of the FEL/OL excitation on the structured Au test sample, providing a quantitative measure of the spatial instrument response function at the sample plane, an example of which is displayed in Fig. 7 ▸. In this image, the chess pattern (black/white colors) can be clearly identified, which was generated using an Hg discharge lamp illuminating several mm^2^ superimposed with the FEL beam (purple/orangs colors) that was focused down to an almost round spot on the target with a diameter of about 50 µm (FWHM). In order to establish the spatial overlap of the FEL and the OL beams, the latter can be easily moved on top of the former in this configuration using the motorized holey laser in-coupling mirror. While it is thereby possible to clearly overlap both beams in real space, the actual size and thus intensity of the OL beam needs to be determined in a different way due to the non-linear nature of the multi-photon imaging process at OL wavelengths, as described elsewhere (Dendzik *et al.*, 2020 ▸).

**Figure 8:**
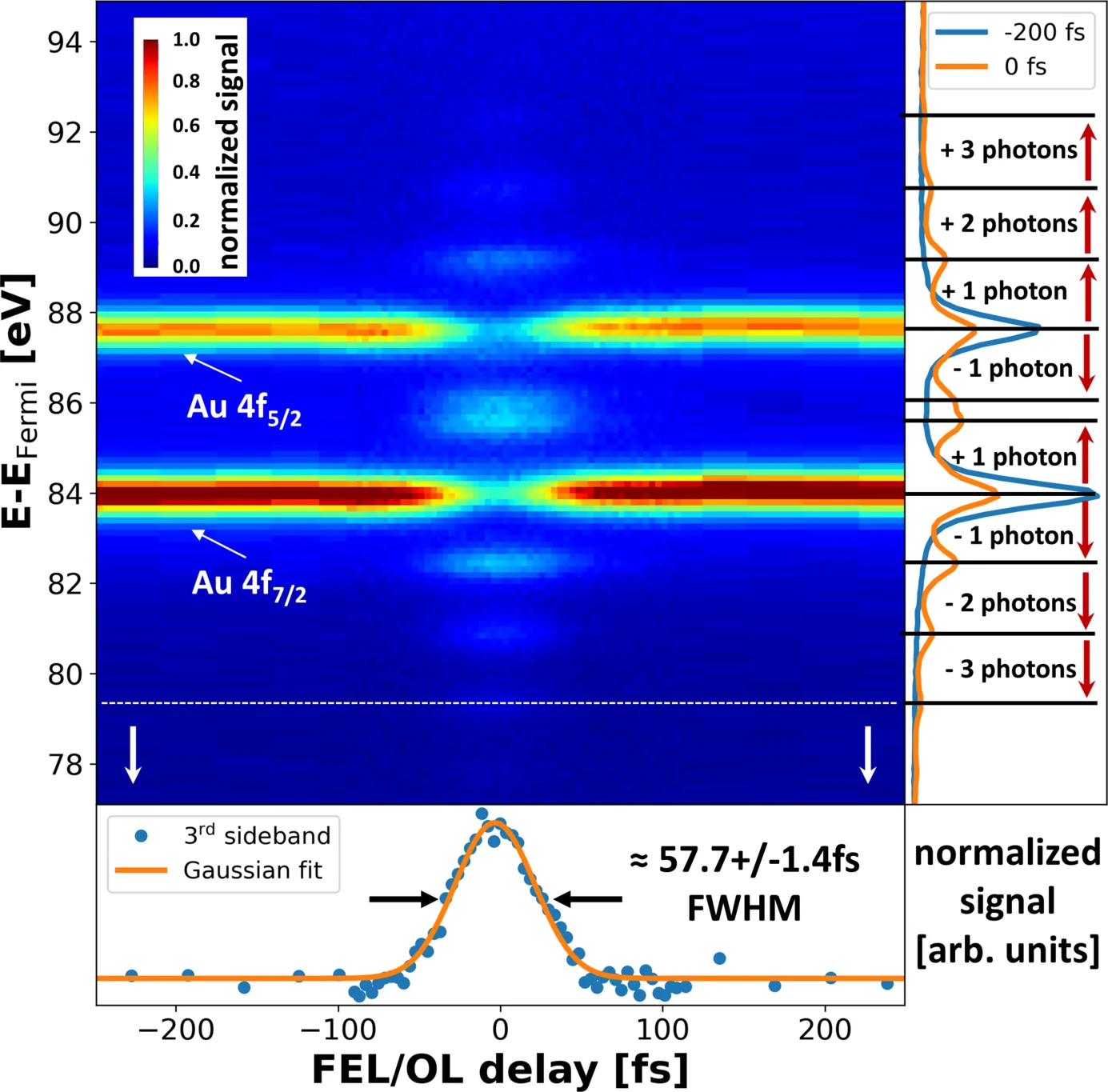
X-ray/optical cross-correlation measurement results: electron spectra of the laser-assisted photoelectric (LAPE) effect on a polycrystalline chess patterned Au reference sample, as a function of the time delay between the FEL and the OL.

In ToF (spectroscopy) mode, the temporal instrument response function can be determined by recording LAPE sidebands from momentum-integrated Au photoelectron spectra as a function of delay between the FEL and OL, an example of which is displayed in Fig. 8 ▸. In the presence of the strong coherent optical field, photoelectrons created by the soft X-ray beam can absorb or emit integer numbers of OL photons. This leads to a ladder of sidebands in the photoelectron spectrum, offset from the main line(s) by integer multiples of the optical photon energy. The visibility and contrast of these sidebands depends sensitively on the temporal overlap between the OL and the FEL, *i.e.* for large negative or positive delays (no temporal overlap), only the main line(s) can be observed, while near temporal overlap, sidebands appear and reach maximum contrast when the laser pulses coincide in time and space at the reference sample. By recording the evolution of the sideband intensity as a function of FEL/OL delay, a high-order cross-correlation of these pulses is obtained (Miaja-Avila *et al.*, 2006 ▸; Wenthaus *et al.*, 2024 ▸).

For the shown imaging and spectroscopy measurements in Figs. 7 ▸ and 8 ▸, the FEL has been tuned to 1 keV (1.24 nm), while the OL pulses were centered at 760 nm (1.63 eV) both operating in the characteristic 10 Hz bunch pattern with an intra-burst repetition rate of about 1.1 MHz. At this soft X-ray photon energy, monochromated in the first diffraction order of the employed low resolution grating (50 lines mm^−1^) and combined with an exit slit opening of 100 µm, experience has shown that FEL pulses with a bandwidth of approximately 351 meV and a duration of about τ_FEL_ = 25 fs are transported to the instruments downstream of the SASE3 undulator (Gerasimova *et al.*, 2022 ▸). In contrast to characterizations of the FEL pulse duration, which are rather based on simulations backed up by isolated measurements due to their complexity, the OL pulse duration can easily be monitored on a daily basis amounting to about τ_OL_ = 45 fs, as determined by a commercial single-shot auto-correlator (FemtoEasy – ROC FS10) on the day of the experimental campaign.

The FEL was operated with linear s-polarization, while the OL was set to linear p-polarization with respect to the plane of incidence. In line with previous studies, a finite out-of-plane field component at the sample arising from the p-polarized OL field is required for efficient sideband formation (Saathoff *et al.*, 2008 ▸). To minimize static space-charge effects induced by the FEL (Pietzsch *et al.*, 2008 ▸), its intensity was carefully adjusted prior to the measurements. The FEL pulse energy at the sample was not measured directly, but estimated from the pulse-resolved energy monitor downstream of the SASE3 undulator, accounting for the transmission of the dedicated gas attenuator and the reflectivity losses of the X-ray optics along the beam path (Gerasimova *et al.*, 2022 ▸). Together with the effective beam area obtained from the FEL spot size measured in PEEM imaging mode on the patterned Au reference sample, this allowed to estimate the on-sample FEL fluence to be around 6.7 × 10^−2^ µJ cm^−2^. This value was optimized to measure the Au 4*f* core levels with a photoionization cross section of σ_Au_ 4*f* (1 keV) ≃ 2 × 10^4^ barn (Yeh & Lindau, 1985 ▸). The FEL fluence for other materials or photon energies is tweaked by adjusting the transmission of the gas attenuator until neither space charge effects nor detector saturation are observed.

Dynamic space-charge effects driven by the OL were suppressed by tuning the on-target fluence to about 1 mJ cm^−2^ (Oloff *et al.*, 2014 ▸), as derived from upstream pulse-energy measurements, the transmission budget of the delivery optics, and the projected focal footprint at the sample, thereby also limiting the maximum sideband order and facilitating the interpretation of the corresponding spectra.

Once the spatial overlap of the FEL and the OL was established in the imaging mode, their temporal overlap was first sought after using the photodiode in the LIN and further narrowed down in spectroscopy mode focusing on the time-resolved Au 4*f* photoemission lines until sidebands were clearly visible, as seen in Fig. 8 ▸. At small and large OL/FEL delays, the photoelectron spectra are dominated by the 4*f*_7/2_ line at 84.0 eV and the 4*f*_5/2_ line at 87.6 eV, corresponding to the two spin–orbit split components of the Au 4*f* core level. As the delays approach time zero, both distinct lines start to vanish while equally spaced sidebands up to the third order appear, both at lower and higher electron energies. To evaluate this cross-correlation, the electron signals of the third sideband order were integrated in a narrow energetic slice around the main peak, normalized by amplitude and fitted with a Gaussian function, as displayed in the bottom graph of Fig. 8 ▸. Thus, a cross-correlation duration of about τ_CC_ = 58 fs was determined at FWHM, which is slightly longer but still close to the results obtained from previous experiments looking at Ne (1*s*) Auger electron spectra at the SQS instrument (Grychtol *et al.*, 2021 ▸; Rivas *et al.*, 2022 ▸). In order to assess the average timing jitter τ_JIT_ between the XFEL and OL pulses from this measurement result, the following easily derived analytical expression assuming the convolution of three Gaussian pulses was used,
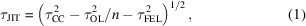
with *n* accounting for the order of the involved non-linearity in the cross-correlation measurement. Considering an OL pulse length of τ_OL_ = 45 fs and an FEL pulse duration of τ_FEL_ = 25 fs, it is possible to deduce an average timing jitter of around τ_JIT_ = 45 fs, right in between the values reported from previous measurements at the European XFEL (Sato *et al.*, 2020 ▸; Grychtol *et al.*, 2021 ▸).

Most importantly, these results clearly demonstrate that this approach is ideally suited for not only setting up an OL/FEL pump–probe experiment at the tr-XPES endstation on a single sample system but also characterizing its spatial as well as temporal resolution *in situ*, *i.e.* the instrument response function.

## 8. Summary

This work outlines the diagnostics and laser infrastructure of the Soft X-ray Port (SXP) at the European XFEL and clarifies the present operational status of its main subsystems. The alignment and delivery chain, optical-laser transport, laser in-coupling optics, and the tr-XPES momentum-microscope endstation are operational and support synchronized soft-X-ray/optical pump–probe experiments. In the current user configuration, the central pump–probe laser provides 800 nm and 1030 nm excitation, while additional wavelength-conversion schemes extend the accessible range toward the ultraviolet and provide a basis for further wavelength extension in future upgrades. The photon arrival-time monitor (PAM) infrastructure is installed at SXP and it is based on a well established spectral-encoding concept. However, under the present conditions at its current location, no detectable XFEL-induced modulation of the transducer membrane has yet been obtained. Consequently, routine single-shot arrival-time correction is not presently available for tr-XPES user operation at SXP. Instead, the currently demonstrated temporal benchmark is provided by *in situ* measurements at the endstation. Using a patterned Au reference sample, PEEM imaging allows the FEL and optical beam overlap and spot sizes at the sample to be characterized, while LAPE sideband spectroscopy yields an effective X-ray/optical cross-correlation of about 58 fs (FWHM) at 1 keV. Together, these measurements define the spatial and temporal instrument response currently achieved for pump–probe tr-XPES experiments at SXP. They also provide a practical basis for planning and interpreting future user experiments and for guiding the development of an upgraded arrival-time monitor optimized for low-fluence operation.

## Figures and Tables

**Table 1:** Operating points of the central pump–probe laser at the SASE3 undulator. For efficient tr-XPES operation, only repetition rates in the MHz regime qualify at SXP

λ (nm)	800	1030
τ_FWHM_	15…300 fs (nearly transform limited)	400 ps and 0.9 ps
		
Intra burst *f*_rep_ (MHz)	*E* (mJ per pulse)	*E* (mJ per pulse)
4.5	0.05	1
2.2	0.15	1.8
1.1	0.25	3.5
0.564	0.5	7
0.282	1	14
0.188	1.5	20
0.112	1.8	36
